# Anesthesia management for percutaneous mitral valve repair in a patient with mitochondrial cardiomyopathy and low cardiac function: a case report

**DOI:** 10.1186/s40981-024-00734-z

**Published:** 2024-08-08

**Authors:** Koichiro Tashima, Masakiyo Hayashi, Takafumi Oyoshi, Jo Uemura, Shinnosuke Korematsu, Naoyuki Hirata

**Affiliations:** Department of Anesthesiology, Kumamoto University Hospital, 1-1-1, Honjo, Chuo-Ku, Kumamoto 860-8556 Japan

**Keywords:** Mitochondrial cardiomyopathy, Remimazolam, Percutaneous mitral valve repair, MitraClip™

## Abstract

**Background:**

Mitochondrial cardiomyopathy occurs when impaired mitochondrial energy production leads to myocardial dysfunction. Anesthetic management in such cases is challenging due to risks of circulatory depression associated with anesthesia and mitochondrial dysfunction induced by anesthetics. Although there are reports of anesthetic management for patients with mitochondrial diseases, there are few reports specifically addressing cardiac anesthesia for patients with mitochondrial cardiomyopathy. We present a case where percutaneous mitral valve repair with MitraClip™ was successfully performed under remimazolam anesthesia in a patient with mitochondrial cardiomyopathy who developed functional mitral valve regurgitation due to low cardiac function and cardiomegaly.

**Case presentation:**

A 57-year-old woman was diagnosed with chronic cardiac failure, with a 10-year history of dilated cardiomyopathy. She was diagnosed with mitochondrial cardiomyopathy 8 years ago. Over the past 2 years, her cardiac failure worsened, and mitral valve regurgitation gradually developed. Surgical intervention was considered but deemed too risky due to her low cardiac function, with an ejection fraction of 26%. Therefore, percutaneous MitraClip™ implantation was selected. After securing radial artery and central venous catheterization under sedation with dexmedetomidine, anesthesia was induced with a low dose of remimazolam 4 mg/kg/h. Anesthesia was maintained with remimazolam 0.35–1.0 mg/kg/h and remifentanil 0.1 μg/kg/min. Noradrenaline and dobutamine were administered intraoperatively, and the procedure was completed successfully without circulatory collapse. The patient recovered smoothly from anesthesia and experienced no complications. She was discharged on the eighth day after surgery.

**Conclusion:**

Anesthesia management with remimazolam appears to be a safe and effective for MitraClip™ implantation in patients with mitochondrial cardiomyopathy.

## Background

Mitochondrial disease is a genetic disorder caused by mutations in genes encoded by either the nuclear or mitochondrial genome. It can be classified as a metabolic disease due to its impact on oxidative phosphorylation pathways, which are involved in ATP production [1]. Mitochondrial disease can cause dysfunction in various organs, including a range of central nervous system disorders, metabolic abnormalities, and endocrinopathies, and is frequently accompanied by increased lactic acid levels [1]. An important point is that the degree of organ dysfunction in mitochondrial disease varies from one case to another. Cardiac involvement is also present in several specific mitochondrial disease syndromes, including Bath, Leigh, Kearns-Sayre syndromes and MELAS (Mitochondrial Encephalomyopathy, Lactic Acidosis, and Stroke-like episodes) syndromes [2]. Mitochondrial cardiomyopathy can be described as a myocardial condition characterized by abnormal heart muscle structure, function, or both, due to genetic defects affecting the mitochondrial respiratory chain [2]. This condition occurs in the absence of concomitant coronary artery disease, hypertension, valvular disease, or congenital heart disease [2].

The determination of the safest and most effective anesthesia method for patients with mitochondrial diseases remains controversial due to potential complications associated with volatile anesthesia [3] or propofol-induced lactic acidosis [4]. Thus, anesthetic management in patients with mitochondrial disease requires careful consideration [5], but there are limited reports on anesthetic management for cardiac surgery in a patient with mitochondrial cardiomyopathy [6].

We report a case where percutaneous mitral valve repair (MitraClip™) was performed under general anesthesia with remimazolam in a patient who developed functional mitral valve regurgitation due to cardiac dilatation caused by mitochondrial cardiomyopathy.

## Case presentation

The patient is a 57-year-old woman (153 cm, 33.4 kg) who has suffered from chronic cardiac failure for 10 years, with underlying conditions of dilated cardiomyopathy. Mitochondrial disease was suspected 8 years ago due to symptoms including diabetes mellitus, high blood lactate levels (19.3 mg/dL), sensorineural hearing loss, heart failure, and proximal muscle-dominant muscular weakness. A muscle biopsy revealed a mitochondrial DNA mutation, leading to a diagnosis of mitochondrial disease (MELAS syndromes). The patient was diagnosed with mitochondrial cardiomyopathy as a cause of chronic heart failure. Although the patient was independent in daily activities and did not exhibit any liver or kidney dysfunction over the past 2 years, the generalized fatigue and shortness of breath associated with mitochondrial cardiomyopathy gradually worsened. Echocardiography revealed a significant decrease in left ventricular function with an ejection fraction of 26%, left ventricular hypertrophy, moderate to severe mitral valve regurgitation, and pulmonary hypertension with an estimated pulmonary artery pressure of 49/19 mmHg. Surgical intervention was deemed necessary due to the limitations of medical therapy alone, and open-heart surgery was considered. However, it was decided to perform percutaneous mitral valve repair with MitraClip™ because the patient was at high surgical risk due to her low left ventricular function.

General anesthesia with remimazolam was planned due to concerns about anesthesia-related complications, including hemodynamic instability. On the day of surgery, no premedication was administered. Prior to the induction of anesthesia, an arterial line was inserted in the left radial artery. After administration of dexmedetomidine for sedation, a central venous catheter with oximetry (Edwards Oximetry CV Catheter™, Edwards Lifesciences, Tokyo) was secured via the internal jugular vein. Anesthesia was induced with remimazolam 4 mg/kg/h, remifentanil 0.1 μg/kg/min, fentanyl 50 μg, and rocuronium 0.6 mg/kg, while noradrenaline 0.03 μg/kg/min and dobutamine 3 μg/kg/min were administered to prevent unexpected circulatory collapse. Tracheal intubation was performed three minutes after loss of consciousness. Hemodynamics remained stable during anesthesia induction.

**Fig. 1:**
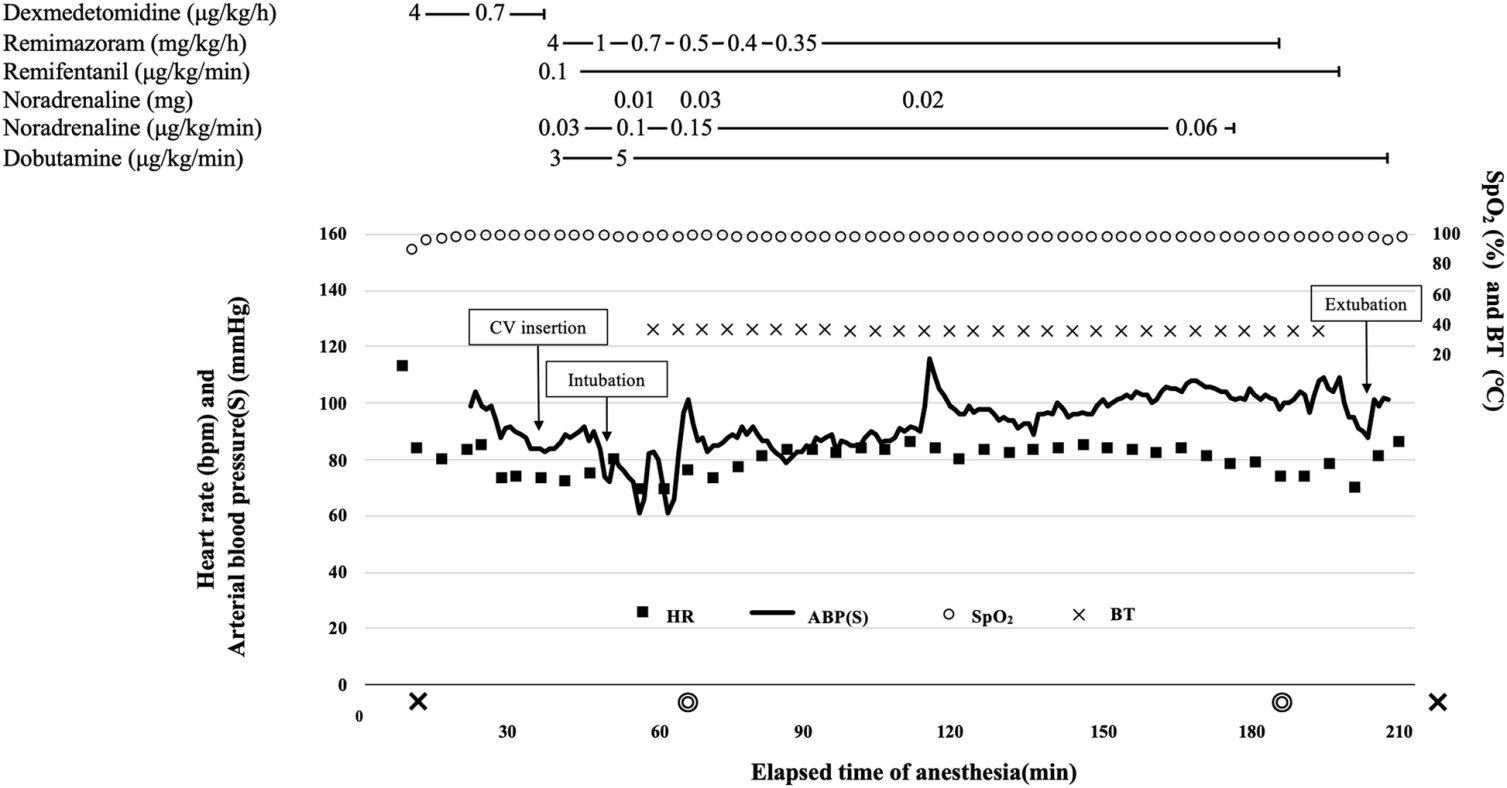
Anesthesia record. × , start and end of anesthesia; ◎, start and end of the operation; HR, heart rate; ABP(S), systolic arterial blood pressure; SpO_2_, saturation of percutaneous oxygen; BT, body temperature

Anesthesia was maintained with continuous administration of remimazolam 0.35–1 mg/kg/h and remifentanil 0.1 μg/kg/min, with the bispectral index (BIS) ranging from 40 to 60. Cardiac performance was monitored by transesophageal echocardiography (TEE) throughout the surgery. Figure 1 shows the anesthesia chart. When blood pressure decreased during surgery, noradrenaline 0.01–0.03 mg was administered. The MitraClip™ was inserted via the left femoral vein and implanted without complications. Intraoperative lactate levels and electrolyte were within the normal range. Operative time was 120 min, anesthesia time was 207 min, and fluid balance was plus 1350 ml.

After the surgical procedures, remimazolam administration was discontinued. Sixteen minutes later, the patient regained consciousness and spontaneous breathing and was extubated. Flumazenil was not administered as the patient’s level of consciousness recovered well, indicated by a Glasgow Coma Scale score of 14 (Eye 3, Voice 5, Movement 6). After extubation, respiratory condition and hemodynamics remained stable, and lactate levels were within the normal range. Dobutamine and noradrenaline were discontinued in the operating room, and the patient was transferred to the intensive care unit. No apparent complications were observed postoperatively, and the patient was discharged from the hospital on the 8th day after the procedure.

## Discussion

We reported the successful anesthesia management using remimazolam for MitraClip™ implantation in a patient with severe mitral valve regurgitation and heart failure due to mitochondrial cardiomyopathy.

To our knowledge, there have only been two reported cases of MitraClip™ for mitochondrial cardiomyopathy [6, 7]. Mitochondrial cardiomyopathy is a rare condition, and it requires particular consideration in anesthetic management [3–5]. Therefore, we believe it is important to accumulate evidence.

Drissen et al. reported that propofol, thiopental, sevoflurane, isoflurane, and halothane were safely used for surgical muscle biopsies in 122 children with mitochondrial defects [8]. Conversely, a study involving 16 children with biopsy-confirmed mitochondrial disease indicated that one child with a complex I mutation and another with Leigh’s disease might be more sensitive to volatile anesthesia [3]. Several experimental studies have shown that volatile anesthetics have depressant effects on the mitochondrial electron transport chain [9, 10]. Additionally, it is known that propofol also affects the electron transport chain in mitochondria [11, 12]. Furthermore, it has been suggested that the effects of propofol on mitochondria are related to the mechanism of propofol infusion syndrome (PRIS) [3, 13]. Several case reports have indicated that patients who developed PRIS had concurrent acquired carnitine deficiency [14] or mitochondrial disease [15, 16]. Although the pathophysiology of PRIS has not yet been clearly elucidated, it is speculated that impaired ATP production in mitochondria may be related [13]. Regarding benzodiazepines, a study demonstrated the effects of midazolam on the mitochondrial electron transport chain using rat liver mitochondria [17]. However, no clinical studies have shown such effects. Importantly, for remimazolam, there have been no reports of effects on mitochondria in both experimental and clinical studies. Furthermore, remimazolam has a milder blood pressure-suppressing effect compared to traditional agents such as propofol [18–20] and volatile anesthetics [21]. It has less impact on autonomic nervous activity [22] and is expected to be safer for use in patients with cardiovascular complications [23, 24]. Therefore, we choose to use remimazolam for general anesthesia in a patient with concomitant mitochondrial cardiomyopathy. In this case, although norepinephrine and dobutamine were used concurrently, perioperative management was successful without complications such as treatment-resistant circulatory depression or acidosis associated with mitochondrial dysfunction.

Percutaneous mitral valve repair, as considered in this case, can be performed under deep sedation or general anesthesia. However, general anesthesia is more advantageous as it allows monitoring by TEE and prevents respiratory artifacts. Furthermore, sedation with dexmedetomidine can potentially cause bradycardia [25], which may worsen mitral regurgitation [26]. Remimazolam is known to have a tendency to increase heart rate [20, 22], and from this perspective, we believe that general anesthesia with remimazolam was a suitable choice for this patient.

In conclusion, anesthesia using remimazolam for percutaneous mitral valve repair was safely managed in a patient with mitochondrial cardiomyopathy. Remimazolam could be a viable anesthetic option for patients with mitochondrial dysfunction and reduced cardiac function.

## Data Availability

Data sharing is not applicable to this article as no datasets were generated or analyzed during the current study.

## Abbreviations

BIS: Bispectral index
ATP: Adenosine triphosphate
DNA: Deoxyribonucleic acid
MELAS: Mitochondrial myopathy, Encephalopathy, Lactic Acidosis, Stroke-like episodes
TEE: Transesophageal echocardiography
